# The Functional Anatomy of the Cornea and Anterior Chamber in Lampreys: Insights From the Pouched Lamprey, *Geotria australis* (Geotriidae, Agnatha)

**DOI:** 10.3389/fnana.2021.786729

**Published:** 2021-12-23

**Authors:** H. Barry Collin, Julian Ratcliffe, Shaun P. Collin

**Affiliations:** ^1^Department of Optometry and Vision Science, University of New South Wales, Kensington, NSW, Australia; ^2^La Trobe Bioimaging Platform, La Trobe University, Bundoora, VIC, Australia; ^3^Oceans Graduate School and Oceans Institute, The University of Western Australia, Crawley, WA, Australia; ^4^School of Life Sciences, La Trobe University, Bundoora, VIC, Australia

**Keywords:** lamprey, cornea, spectacle, iris, stroma, sutures, annular ligament, pectinate ligament

## Abstract

Extant lampreys (Petromyzontiformes) are one of two lineages of surviving jawless fishes or agnathans, and are therefore of critical importance to our understanding of vertebrate evolution. Anadromous lampreys undergo a protracted lifecycle, which includes metamorphosis from a larval ammocoete stage to an adult that moves between freshwater and saltwater with exposure to a range of lighting conditions. Previous studies have revealed that photoreception differs radically across the three extant families with the Pouched lamprey *Geotria australis* possessing a complex retina with the potential for pentachromacy. This study investigates the functional morphology of the cornea and anterior chamber of *G. australis*, which is specialised compared to its northern hemisphere counterparts. Using light microscopy, scanning and transmission electron microscopy and microcomputed tomography, the cornea is found to be split into a primary spectacle (dermal cornea) and a scleral cornea (continuous with the scleral eyecup), separated by a mucoid layer bounded on each side by a basement membrane. A number of other specialisations are described including mucin-secreting epithelial cells and microholes, four types of stromal sutures for the inhibition of stromal swelling, abundant anastomosing and branching of collagen lamellae, and a scleral endothelium bounded by basement membranes. The structure and function of the cornea including an annular and possibly a pectinate ligament and iris are discussed in the context of the evolution of the eye in vertebrates.

## Introduction

Lampreys (Petromyzontiformes) and hagfishes (Myxiniformes) are the extant representatives of the agnathan (jawless) stage in vertebrate evolution. There are 42 known extant species of lamprey; 37 species belonging to the family Petromyzontidae restricted to the northern hemisphere (Hubbs and Potter, 1971; Potter et al., 2015) and five species separated into two families in the southern hemisphere, with Mordaciidae containing three species and Geotriidae represented by two species, *Geotria australis* and the recently reinstated *Geotria macrostoma* (Potter and Strahan, 1968; Riva-Rossi et al., 2020). Phylogenetic analyses suggest that northern hemisphere lampreys are the most derived and of the two southern hemisphere genera (*Geotria* and *Mordacia*), *Mordacia* is the most basal (i.e., most similar to the ancestral stock; Potter et al., 2015), but the issue is not fully resolved (Evans et al., 2018).

Lampreys have a protracted larval phase, where the larvae are microphagous and photophobic (Hardisty and Potter, 1971a,b), burrowing in freshwater rivers for many years, before they undergo a radical metamorphosis to become adults. Anadromous species such as the Pouched lamprey, *Geotria australis* then migrate downstream to enter their marine phase, where they are found in high numbers throughout the austral summer in the cold and clear waters surrounding South Georgia (Potter et al., 1979; Prince, 1980). *G. australis* form a substantial component of the diet of the grey-headed albatross, *Diomedea chrysostoma* (Prince, 1980), which forage on parasitised teleost fishes (Hardisty, 1979; Potter et al., 1980) in the brightly-lit surface waters. As a means of camouflage, both downstream and marine phase *G. australis* adopt a countershading colouration with a luminance gradient from dark (blue) dorsal to pale (silver) ventral pigmentation to enhance the match between the radiance of the body and that of the background from different viewing angles. Upon reaching maturity, *G. australis* then return to their natal freshwater river and migrate upstream, where they spawn and ultimately die (Potter et al., 1983).

The image-forming eyes of *G. australis* have been found to be the most specialised of all lampreys examined, at least with respect to photoreception. In contrast to the northern hemisphere (holarctic) lampreys and members of the only other southern hemisphere family of lampreys (Mordaciidae), the pouched lamprey possesses five types of photoreceptors (based on both morphology, spectral sensitivity and visual opsin expression, Collin et al., 2003a,b; Warrington et al., 2020), providing the potential for pentachromatic vision under some light conditions throughout its protracted life cycle. The eyes of members of the holarctic Petromyzontidae possess two photoreceptor types (a cone-like and a rod-like, Holmberg et al., 1977; Ishikawa et al., 1987), while the Mordaciidae possess only a single type of rod-like photoreceptor (Collin and Potter, 2000). Although a non-spherical lens to mediate variable focus and a split cornea have previously been reported in *G. australis* (Collin et al., 1999), there are no detailed analyses of the cornea in any southern hemisphere species of lampreys.

Both Walls (1942) and Duke-Elder (1958) describe the eyes of many species of northern hemisphere (holarctic) lampreys to be flattened antero-posteriorly with the cornea split into dermal and scleral components separated by a delicate layer of mucoid tissue. A prominent feature of these eyes is a transparent “window” in the dermis overlying the eye that forms the dermal cornea. The mucoid layer allows the eye to rotate beneath the dermal goggle. The arrangement of a “fixed transparent structure separate from the globe underneath which the eye is free to rotate” is defined as a primary spectacle (Treviranus, 1820; Franz, 1934), which is derived from the surface ectoderm. This arrangement is different from a secondary spectacle, which is formed by the development of a transparent area of the eyelids by the edge to edge fusion of two lids that have become transparent to form a fixed spectacle (Hein, 1913; Franz, 1934; Walls, 1942). The lens is close to the scleral cornea, which is continuous with the sclera of the eyecup. The tendon of a large cornealis muscle located outside and caudal to the eyecup inserts into the posterior edge of the dermal cornea, which, upon contraction, pulls the dermal and scleral corneas and the lens, toward the retina to accommodate from myopia to emmetropia (Walls, 1942).

The split corneas of the Northern hemisphere lampreys including the Sea lamprey, *Petromyzon marinus* (Van Horn et al., 1969a,b**;** Pederson et al., 1971) and the European river lamprey, *Lampetra fluviatilis* (Dickson and Graves, 1981) have previously been described. The dermal cornea is thick with a layer of stratified squamous epithelial cells overlying a stroma of collagen lamellae, many of which are linked by sutural fibres that inhibit swelling and prevent the loss of transparency during environmental changes (Van Horn et al., 1969b; Pederson et al., 1971). A mucoid layer bounded by mesodermal layers of cells, overlies a scleral cornea with a stroma that lacks fibroblasts and a posterior layer of endothelial cells. Dickson and Graves (1981) also provide an ultrastructural description of the eye in the Sea lamprey, *P. marinus* including the anterior chamber, iris and lens.

There are only a few studies of the cornea in southern hemisphere lampreys, namely, the Pouched lamprey, *Geotria australis* (Collin et al., 1999; Collin and Collin, 2000a,^2006^) and the Shorthead lamprey, *Mordacia mordax* (Collin and Collin, 2000a,^2006^; Collin and Potter, 2000), with these studies focussing only on light and scanning electron microscopical examination of the ultrastructure of the corneal epithelial surface. One publication used transmission electron microscopy to briefly describe the ultrastructure of the cornea of the ammocoete larva of *Geotria australis* (Meyer-Rochow and Stewart, 1996).

The aim of this study is to describe the functional morphology of the cornea and anterior chamber in the Pouched lamprey, *Geotria australis*. This anadromous species represents one of only two species in the family Geotriidae and, compared to the other two families of lampreys, possesses a specialised visual system with the potential for pentachromacy over a range of light conditions throughout its lifecycle. It is hoped that this study will improve our understanding of the evolution of the cornea and its role in vision.

## Materials and Methods

### Source of Animals

Ten recently-metamorphosed, downstream migrating young adults of *Geotria australis* (Gray, 1851) (75–110 mm in total length) were collected from streams and rivers in south-western Australia using an electric fish shocker. All collection, holding and experimental procedures followed the guidelines of the National Health and Medical Research Council (NHMRC) - Australian Code of Practice for the Care and Use of Animals for Scientific Purposes, in accordance with The University of Western Australia Animal Local Ethics protocol (Approval Numbers: RA/3/100/917 and RA/3/100/1220). Three upstream migrants of *G. australis* at approximately 2–3 months after they had commenced their spawning run (50–60 cm in total length) were also captured using fish traps at Meadowbank dam in the Derwent River, Tasmania (Inland Fisheries Service Permit Number: 2011-32) and transported to The University of Western Australia (Department of Fisheries Translocation Permit Number: 871/11). Animals were kept in aquaria in a temperature-controlled room that was maintained at 10–14°C with a 12 h:12 h light:dark cycle. While maintained in aquaria, the head and eyes of both downstream and upstream migrants were photographed using a Nikon digital camera (D5600) (Collin and Collin, 2021b).

Animals were euthanised by immersion in a 0.5 mg ml^–1^ solution of tricaine methanesulfonate (MS-222; Sigma-Aldrich, Australia) buffered with an equal concentration of NaHCO_3_ (Ajax Finechem, Australia) in the light phase of the light/dark cycle.

### Light and Electron Microscopy

Enucleated eyes, including the dermal cornea, were immersion-fixed in Karnovsky’s fixative (2.5% (w/v) glutaraldehyde (ProSciTech, Australia), 2% (w/v) PFA and 1% dimethyl sulphoxide (DMSO; Sigma-Aldrich, Australia) in 0.1 M sodium cacodylate buffer (ProSciTech, Australia), pH 7.4) and stored at 4°C. Eyes were post-fixed in 1% (w/v) osmium tetroxide (Sigma-Aldrich, Australia) in 0.13M Sorenson’s buffer (0.13M Na_2_HPO_4_ (Millipore Sigma, Australia), 0.13 M KH_2_PO_4_ (Sigma-Aldrich, Australia), pH 7.4) for about 2 h. The tissue was rinsed in 0.13 M Sorenson’s buffer, dehydrated through a graded series of ethanols (25, 50, 70, 80, 95, and 100%) followed by treatment with propylene oxide (VWR, Australia), before infiltration with araldite (ProSciTech, Australia) or Spurrs (Sigma-Aldrich) resin using a tissue processor (Leica-Reichert Lynx). The samples were then cured at 60°C overnight.

Transverse and tangential (semi-thin) sections (1–2 μm) were cut using an American Optical rotary microtome and a glass knife. Sections were mounted on subbed slides, stained with Toluidine blue, examined using a BH-2 Olympus compound light microscope and photographed on an Olympus DP-30 digital camera fitted with a trinocular C mount. Ultrathin sections (70–90 nm) were cut using a diamond knife (DiATOME 45°), collected on copper grids with 200 mesh or rectangular 75/300 mesh (ProSciTech, Australia) and stained using lead citrate (Reynolds, 1963) and uranyl acetate according to Collin et al. (1999). Sections were examined on a Philips 410 transmission electron microscope operated at 80 kV and photographed using Kodak Technical Pan black and white film rated at 100 ASA, or on a Jeol JEM-2100 LaB_6_ TEM operated at 80 kV and photographed on a Gatan Orius SC 200 CCD.

Following post-fixation of the eyes in osmium tetroxide in 0.1 M cacodylate buffer and dehydration in a graded series of alcohols (30, 50, 70, 90, 95, and 100%), ocular tissue was critical point-dried in either a Polaron (Watford, United Kingdom) or a Tousimis (Labtech, United Kingdom) critical point dryer and mounted on 10-mm aluminium stubs with double-sided tape. Once the two corneas were dissected free of the globe at the limbus, they were carefully separated under a dissecting microscope (Nikon SMZ745T). Both corneas were then hemisected so that half of the cornea was inverted and both sides were displayed in order to ensure both anterior and posterior surfaces were differentiated. Selected mounted corneal pieces were examined using one of two methods: (1) Corneal tissue was coated with 12–15 nm of gold-palladium in a Polaron sputter coater, placed in an oven at 40°C overnight before being examined using a JEOL field emission scanning electron microscope operated at an accelerating voltage of 3 kV; and (2) Corneal tissue was coated with 5 nm platinum in a Safematic CCU-010 HV sputter coater (Microscopy Solutions, Australia) and examined using a Hitachi SU7000 field emission SEM operated at an accelerating voltage of 5 kV, equipped with backscattering secondary electron and STEM detectors. Results were recorded both on 35 mm film and digitally. The Hitachi SU7000 FESEM multi zig-zag function was used for automated wide area montaging in order to examine the extent of the insertion of the annular ligament into the scleral cornea.

### Micro Computed Tomography

A block containing the eye of a downstream migrant, prepared as above for TEM imaging was used for microCT imaging. Micro X-ray Computed Tomography (μXCT) measurements were carried out using an Xradia^©^ micro XCT200 (Carl Zeiss X-ray Microscopy, Inc.). This uses a microfocus X-ray source with a rotating sample holder and an imaging detector system consisted of coupling objective lens and CCD camera. The source consists of a closed x-ray tube with the tube voltage of 40 kV and a peak power of 10W. One data acquisition set consisted of 361 equiangular projections over 180 degrees. The exposure time was 1 s for each projection. The tomographic scan involved rotating the sample whilst recording transmission images on the CCD. Each projection image was corrected for the non-uniform illumination in the imaging system, determined by taking a reference image of the beam without sample. A cone beam filtered back-projection algorithm is used to obtain the 3D reconstructed image. The final three-dimensional reconstructed image size was 512 × 512 × 512 voxels with the voxel size of 7 μm along each side and Field of View (FOV) of (3.5 mm)^3^. A lateral image of the eye is presented to show the location and size of the annular ligament with respect to other ocular features.

### Quantitative Analyses

Measurements of both surface (using SEM) and internal (using TEM) corneal features in left and right eyes were performed on digital images using the Photoshop calibration tool (Version 20.0.4). Although all features of the cornea and anterior chamber were examined in both phases (downstream and upstream migrants), all morphometric measurements presented are only for the downstream individuals. The ultrastructure of the cornea and anterior chamber was examined in both downstream and upstream stages in order to assess whether there were any major morphological changes during the anadromous adult phase of the lifecycle of this species, other than overall changes in eye size. Since there were no major changes, we restricted our morphometric analyses to only the eyes of downstream migrants. For statistical reasons, we targetted downstream migrants, since they were all captured just prior to entering the marine phase and had a comparable eye size. Between 20 and 50 examples of each corneal feature were measured (± standard deviation) and dimensions were compared using a two-tailed t-test for independent variables. Measurements were performed on both left (*n* = 3) and right (*n* = 4) eyes of seven individual downstream migrants, but all features were not found to be significantly different, as has been found for corneal features in a range of other vertebrate eyes (Nam et al., 2006; Werther et al., 2017; Collin and Collin, 2021b). Therefore, the data for left and right eyes were pooled. The mean and standard deviation for each corneal component are presented in Table 1. No attempt was made to assess the degree of shrinkage in our study in order to allow direct comparison to be made with measurements derived using previously published electron micrographs using similar methods. However, due to the different methods of histological processing, it is expected that the same corneal features measured using scanning and transmission electron microscopy may differ slightly. Shrinkage of around 30–40% is expected following fixation and resin embedding for transmission electron microscopy (Hayat, 1986). Therefore, a correction factor should be applied to the data presented to give an estimate of the *in vivo* tissue and cell dimensions. All comparisons of the arrangement and morphometric analyses of components of the cornea and anterior chamber in other species are with comparable adult phases unless otherwise indicated.

**TABLE 1:** Summary of the components of the dermal and scleral corneas and their dimensions in the Pouched lamprey, *Geotria australis* listed from anterior to posterior.

Corneal Region	Central	Peripheral
**Dermal cornea**		
Epithelium	24.4 ± 4.1 μm	61.9 ± 16.1 μm
Basement membrane	140 ± 31 nm	140 ± 31 nm
Bowman’s layer	Not present	Not present
Stroma (∼100 lamellae)	24.4 ± 3.1 μm	110.1 ± 34.7 μm
Basement membrane	Not present	Not present
Monocellular layer	885 ± 68 nm	885 ± 68 nm
Basement membrane	20.7 ± 7 nm	20.7 ± 7 nm
**Total dermal cornea**	49.85 μm	173.05 μm
Mucoid layer	Present	Present
**Scleral cornea**		
Basement membrane	25 ± 3 nm	25 ± 3 nm
Monocellular layer	978 ± 29 nm	978 ± 29 nm
Basement membrane	Not present	Not present
Stroma (∼20 lamellae)	4.75 ± 0.64 μm	15.6 ± 4.5 μm
Basement membrane	86 ± 15 nm	86 ± 15 nm
Monocellular layer (endothelium)	707 ± 45 nm	707 ± 45 nm
Basement membrane	37 ± 12 nm	37 ± 12 nm
**Total scleral cornea**	6.58 μm	17.83 μm
Annular ligament	Not present	Zero to 135 μm

## Results

### Dermal Cornea

**FIGURE 1:**
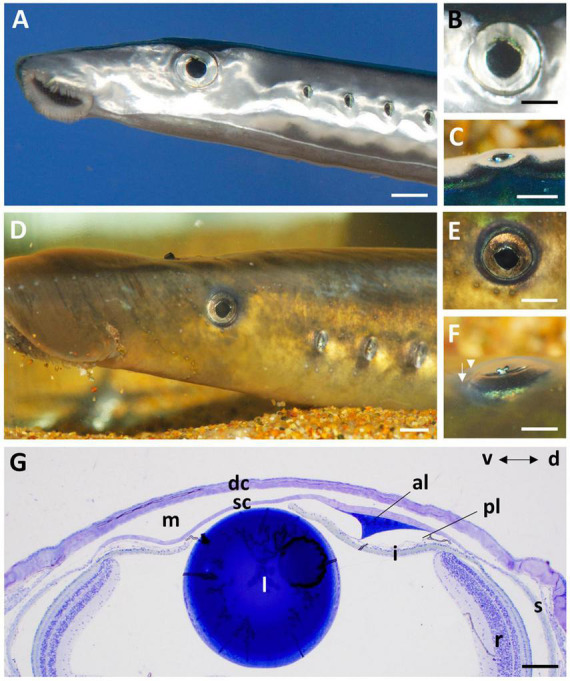
The eye and anterior chamber of the pouched lamprey, *Geotria australis* in the downstream **(A–C)** and upstream **(D–F)** migrant stages. The eyes are laterally-placed in both stages with a near-circular pupil **(A,B,D,E)**, an iris with a ruffled edge on the dorsal border **(B,E)** and a y-shaped iridial operculum that projects laterally **(C,F)**. Note the differentiation of the dermal (arrowhead) and scleral (arrow) corneas in panel **(F)**. **(G)** Transverse section of the cornea and anterior chamber of the eye of a downstream migrant showing the close proximity of the lens (l) to the scleral cornea (sc) and the prominent annular ligament (al) in the dorsal (d) region. dc, dermal cornea; i, iris; m, region occupied by a mucoid layer; pl, possible pectinate ligament; r, retina, s, sclera; v, ventral. Scale bars: 2 mm **(A)**; 1 mm **(B)**; 2 mm **(C)**; 5 mm **(D)**; 3 mm **(E)**; 2 mm **(F)**; 0.2 mm **(G)**.

The cornea of the Pouched lamprey (*Geotria australis*) possesses a primary spectacle (dermal cornea) and a scleral cornea separated by a narrow mucoid layer, which widens toward the periphery (Figure 1). Using light and transmission electron microscopy, the thickness of the dermal cornea was 49.85 μm in the centre with the epithelium (24.4 ± 4.1 μm) and stroma (24.4 ± 3.1 μm) being of equal thickness (Figure 1G). In the periphery, there is a three-fold increase in thickness to 173.05 μm, with the dermal stroma occupying almost two thirds of the total corneal thickness (Table 1). In the caudal periphery of the dermal cornea, there is a dorso-ventral seam of connective tissue, which indicates the insertion of the tendon of the caudal cornealis muscle. The cornealis muscle is located extra-orbitally and just beneath the dermis of the head. A suspensory ligament and an intraocular retractor muscle are absent, leaving the lens to be supported only by the vitreous humour, the iris, scleral cornea and a thin fibrous membrane extending from the lens equator to the ora retinalis (Gustafsson et al., 2010).

**FIGURE 2:**
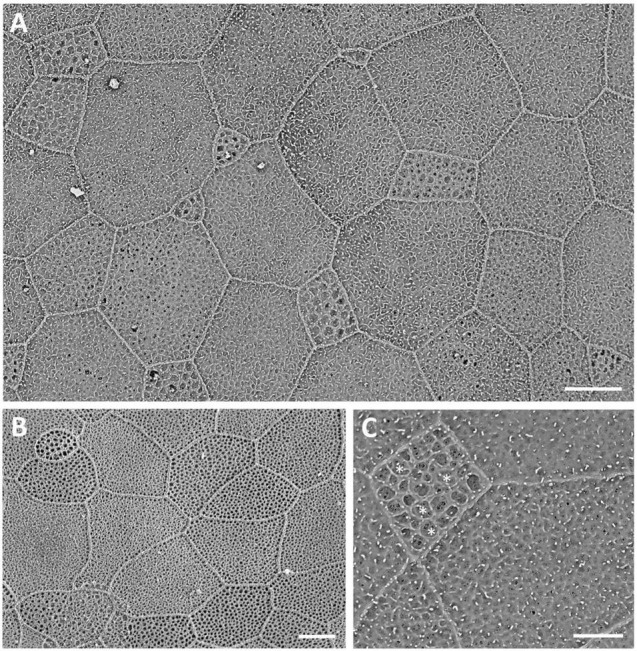
Surface ultrastructure of the dermal cornea viewed using scanning electron microscopy **(A–C)**. The surface of the cornea shows an array of polygonal epithelial cells, which range in size. Projecting from the surface of the epithelial cells are microridges and microvilli, which surround mucus-filled microholes. Note the largest holes occur within the smallest cells, each of which contains a dense accumulation of mucous [asterisks in panel **(C)**]. Scale bars: 6 μm **(A)**; 5 μm **(B)**; 2.5 μm **(C)**.

Viewed using scanning electron microscopy (SEM), the surface of the dermal cornea is covered by polygonal, mainly rounded hexagonal and pentagonal epithelial cells (Figure 2). The superficial epithelial cells are covered by numerous microprojections (both microvilli and interweaving microplicae) with a mean width of 115 ± 6 nm and a density of 5,511 ± 2,025 cells/mm^–2^ (density previously published by Collin and Collin, 2000a,^2006^). Between the microplicae, are numerous holes or pits (microholes), which vary in size up to 660 nm in diameter, with a previously published mean diameter of 395 ± 15 nm (Collin and Collin, 2006). However, we remeasured the diameter of the corneal holes using SEM and found that the size (diameter) of the holes was dependent on the size of the cell, with the smallest cells having the largest holes and the larger cells having the smallest holes, while the largest cells had no holes (Table 2 and Figures 2A–C). In addition, the microhole maximum diameter as a percentage of the cell maximum diameter decreased from 11.34 ± 1.84% for the small cells (up to 8 μm) to 1.38 ± 0.45% for the large cells (15–19 μm) (Table 2).

**TABLE 2:** A comparison between the diameter of the holes in the epithelial cells of the cornea of the Pouched lamprey, *Geotria australis* and the diameter of the corneal epithelial cells.

	Diameter of holes	Comparison	Significance	% of cell diameter*
Small cells (up to 8 μm)	457 ± 89 nm	Small versus medium	*p* < 0.00001	11.34 ± 1.84
Medium cells (9–14 μm)	317 ± 55 nm	Medium versus large	*p* < 0.00001	3.25 ± 1.70
Large cells (15–19 μm)	196 ± 38 nm	Small versus large	*p* < 0.00001	1.38 ± 0.45
Largest cells (>20 μm)	No holes		All significant	

*Microhole maximum diameter as a percentage of the cell maximum diameter.

**FIGURE 3:**
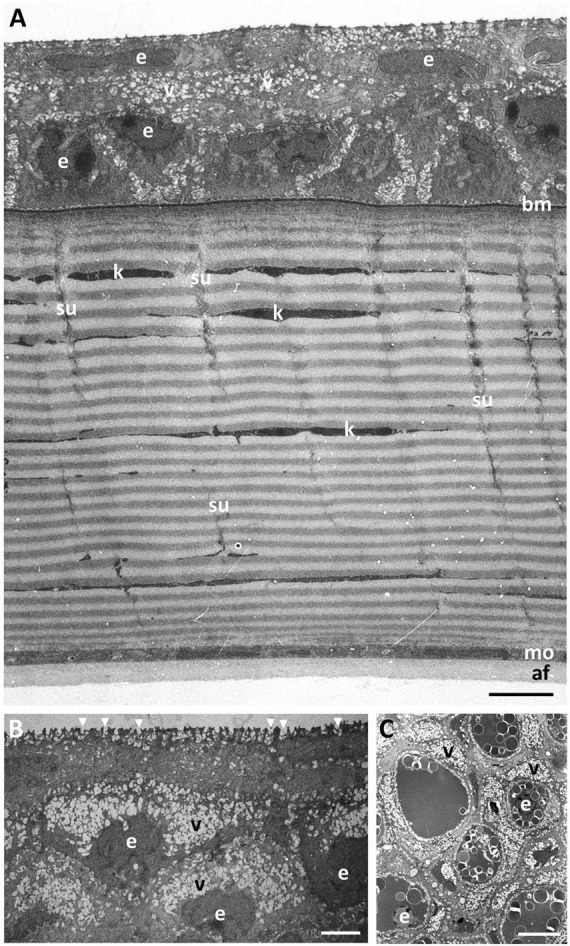
Ultrastructure of the dermal cornea. **(A)** Transverse section of the full thickness of the dermal cornea showing the layers of stratified squamous epithelial cells (e) overlying a basement membrane (bm), a stroma comprised of up to 100 lamellae of collagen fibrils, and a monolayer of cells (mo). The posterior surface of the dermal cornea is lined with an amorphous aggregation of fibres (af). Note the gradation of cytoplasmic vacuoles (v) with the highest concentration in the more superficial epithelial cells with the high number of microholes at the surface (arrowheads) **(B)**. Individual collagen lamellae are interrupted by keratocytes (k) and vertically-oriented sutures (su). **(C)** Transverse section through the limbal region of the dermis showing large epithelial cells containing aggregations of spherical vesicles resembling lipids and high concentrations of vacuoles. Scale bars: 10 μm **(A)**; 2.5 μm **(B)**; 10 μm **(C)**.

**FIGURE 4:**
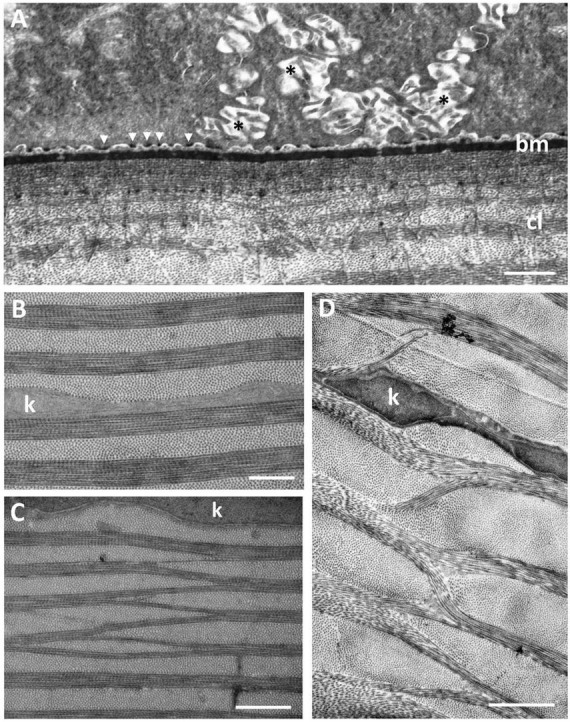
**(A)** Posterior region of the corneal epithelium showing the dense basement membrane attached to the basal epithelial cells via hemidesmosomes (arrowheads). Note the complex interdigitations of the adjacent cell membranes (asterisks). The anterior region of the corneal stroma is comprised of a series of corneal lamellae (cl) containing only one collagen fibril. Bowman’s layer is absent. **(B)** Middle region of the stroma of the central dermal cornea showing the collagen lamellae with bundles of collagen fibrils with alternating orientations. **(C,D)** Branching and anastomosing of the collagen lamellae in the peripheral cornea showing bundles of collagen fibrils running obliquely between lamellae with the same orientation, interrupted by keratocytes (k). Scale bars: 6 μm **(A)**; 0.5 μm **(B)**; 1 μm **(C)**; 2 μm **(D)**.

Viewed using transmission electron microscopy (TEM), the superficial epithelium has five or six layers of stratified squamous cells (Figure 3A). The shape and arrangement of these cells appears to fit the criteria for scutoids as presented by Gómez-Gálvez et al. (2018). The intermediate (wing) epithelial cells immediately below the superficial cells are flatter and wider (up to 14.5 μm) with elongated nuclei, and contain high numbers of vacuoles, which are almost exclusively aggregated on the side of the nucleus nearer to the epithelial (anterior) surface (Figure 3B). The mean diameter of these intracellular vacuoles (430 ± 191 nm) is not significantly different (*p* = 0.463) from the diameter of the surface holes. These vacuoles contain amorphous material, presumably mucus and are arranged in cylindrical formations extending up to 2 μm beneath the surface of the epithelium and are separated by cell cytoplasm containing fine filaments (Figure 3B). The mean diameter of the surface holes is 403 ± 128 nm, which is not significantly different from the mean diameter of the surface holes observed using SEM. Adjacent to these holes, and above the surface of the epithelium, sit many small globules of mucus, with some globules appearing to be in the process of release through the surface epithelial holes. There are also some thin flattened cells up to 25 μm in width, with elongated nuclei, cytoplasm devoid of vacuoles and a surface with small microvilli but without holes. All epithelial cells interdigitate with one another and are bound together with desmosomes with a density of around 7.5 desmosomes per square micron (Figure 4A). In the limbal region and continuing into the skin, the epithelial cells are large (up to 24 μm in diameter), each containing a round nucleus with a nucleolus and up to 20 vesicles containing an amorphous material resembling lipid (Figure 3C). When observed using transmission electron microscopy, the basal epithelial cells are tall and columnar with large round nuclei, cytoplasm devoid of vacuoles and a diameter of 5.4–6.5 μm. The basal epithelial cells are attached to a dense basement membrane (with a thickness of 140 ± 31 nm) by numerous hemidesmosomes. There are some areas where the basement membrane is less dense or absent and these gaps are 98.2 ± 11.4 nm wide (Figure 4A).

The dermal stroma does not possess a Bowman’s layer and there is no evidence of anchoring fibrils or anchoring plaques in the region of the basement membrane (Figure 4A). The stroma consists of approximately 100 lamellae of collagen fibrils, which have a mean diameter of 28.5 ± 4 nm and a D-periodicity of 59.6 ± 3.3 nm. The direction of the collagen fibrils in each lamella is approximately at right angles to the adjacent lamellae (Figures 3A, 4B). However, the lamellae are not of equal thickness. In the anterior stroma, each lamella consists of only one collagen fibril, while each lamella in the middle and posterior regions of the stroma contain around 10 and 3 collagen fibrils, respectively (Figures 3A, 4A). The variation in mean lamellar thickness throughout the depth of the stroma is 47.0 ± 4.8 nm (anterior 10 lamellae), 337.5 ± 44.4 nm (the central 10 lamellae) and 99.9 ± 16.1 nm (the posterior 10 lamellae). There are a few flattened cells (keratocytes) scattered throughout the stroma and these lie between the lamellae (Figure 4B). No polymorphonuclear leucocytes or pigment cells are present within the stroma.

The stroma of the dermal cornea possesses several types of specialisation with respect to the arrangement of collagen fibrils, namely extensive branching and anastomosing of the collagen lamellae and several types of vertical sutures. With respect to collagen branching, this may consist of single or multiple collagen fibrils running obliquely between two collagen lamellae with the same orientation of fibrils (Figures 4C,D). Although present in some central areas of the dermal cornea, branching and anastomosing is predominantly in the peripheral cornea. There are several types of “vertical sutures” present in the dermal corneal stroma, although they appear to be present only in the central cornea (Figure 3A).

**FIGURE 5:**
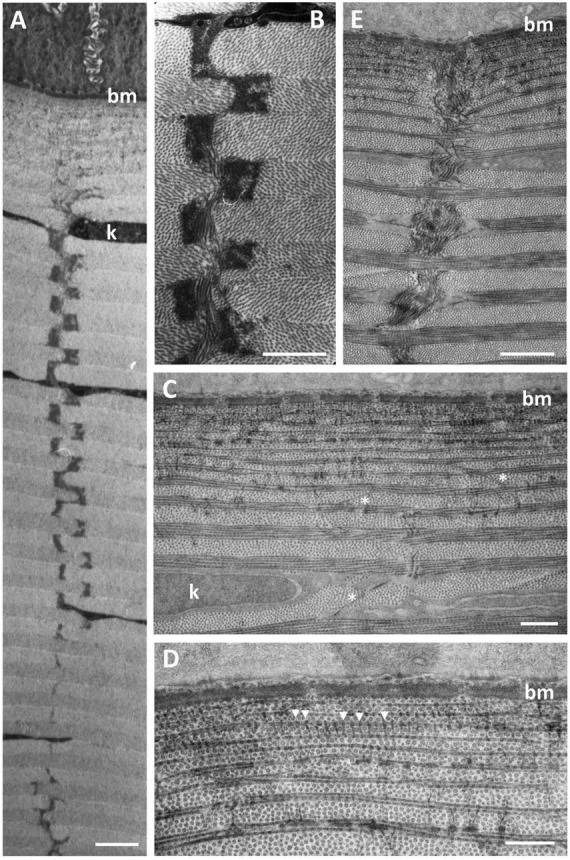
Ultrastructure of vertical sutures in *G. australis*. **(A,B)** Type 1 Amorphous vertical sutures showing the alternating osmiophilic plaques of presumably proteoglycans piercing many collagen lamellae throughout the depth of the dermal corneal stroma. **(C,D)** Anterior region of the dermal stroma showing Type 2 (single collagen fibrils extending between two collagen lamellae of the same orientation, between arrowheads) and 3 (single collagen fibrils extending vertically or at random angles across two or more collagen lamellae, asterisks) vertical sutures. **(E)** A Type 4 vertical suture, which consists of a large bundle of collagen fibrils extending vertically through multiple collagen lamellae. Scale bars: 10 μm **(A)**; 7 μm **(B)**; 0.5 μm **(C)**; 0.5 μm **(D)**; 15 μm **(E)**.

Four (sub) types of suture are characterised on the basis of the number of collagen fibrils, their orientation and stromal depth: (1) *Amorphous vertical sutures*, consisting of collections of amorphous material, probably proteoglycans (Alanazi et al., 2015), and occasional collagen fibrils, extending over two or more collagen lamellae (Figures 5A,B); (2) *Small vertical sutures*, consisting of single collagen fibrils, approximately 25 nm wide and 75 nm long, extending between two lamellae of the same collagen fibril orientation (Figures 5C,D). These sutures appear to occur in only the most superficial lamellae, where each lamella consists of only one or two collagen fibrils. The position of these sutures appears to correspond with the periodic darkly-stained regions of the collagen fibrils (Figure 5D). Collectively, they resemble a “picket fence”; (3) *Single fibre vertical sutures*, consisting of individual collagen fibrils running vertically or at random angles across two or several collagen lamellae, up to 2.2 μm in length but probably extend over greater distances in other planes (Figure 5C). These sutures occur only in the anterior region of the central cornea and do not constitute a Bowman’s layer; (4) *Large vertical sutures*, consisting of large bundles of collagen fibrils extending vertically through most (perhaps all) of the dermal stroma. The bundles of fibrils appear to weave their way through the corneal lamellae (Figure 5E). Associated with the collagen bundles are large deposits of usually darkly-staining amorphous material, probably proteoglycans (Alanazi et al., 2015). When observed using the light microscope, these large sutures may take on a square saw-toothed appearance (Figures 3A, 5A,B).

**FIGURE 6:**
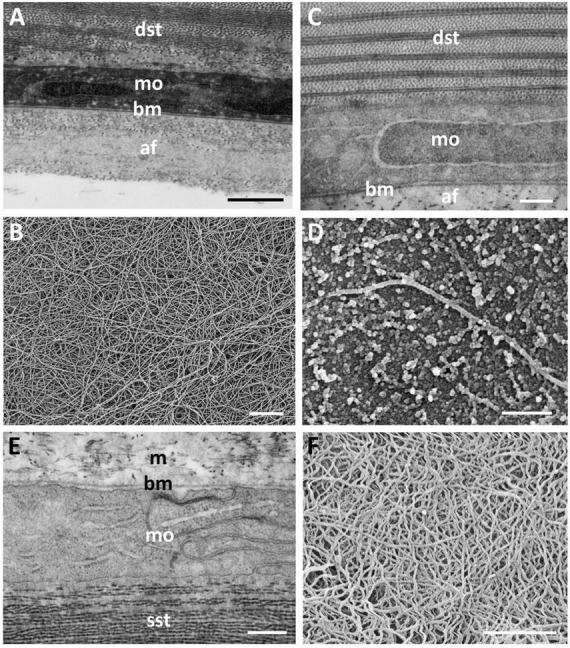
Ultrastructure of the lining of the mucoid layer separating the dermal and scleral corneas. **(A)** Transmission electron micrograph of the posterior region of the central dermal cornea, which consists of the stroma (dst), a monolayer of cells (mo) and a basement membrane (bm). Posterior to the basement membrane lies a collection of amorphous material with scattered fibres (af) resembling thin collagen fibrils, which are shown using scanning electron microscopy (SEM) in panel **(B)**. In peripheral cornea, the scattered fibres of the mucoid layer are less dense **(C)**, where the surface of the basement membrane is revealed using SEM **(D)**. **(E)** The anterior surface of the scleral cornea underlying the mucoid layer (m) is covered by a basement membrane and another monolayer of cells, which in turn overlies the scleral stroma (sst). The anterior surface of the scleral cornea is covered by a dense tangle of fibres as viewed using SEM **(F)**. Scale bars: 1 μm **(A)**; 1 μm **(B)**; 0.5 μm **(C)**; 0.25 μm **(D)**; 0.5 μm **(E)**; 0.8 μm **(F)**.

Posterior to the dermal stroma is a single layer of cells (endothelium) with large nuclei and an overall thickness of 885 ± 68 nm (Figures 6A,C). There is a continuous basement membrane (20.7 ± 7 nm thick) extending over the internal (mucoid) side of the posterior cellular monolayer, with no microprojections or cilia. There is no basement membrane between the stroma and the posterior cellular layer.

### Mucoid Layer

Between the dermal and the scleral corneas, there is a mucoid layer consisting of amorphous material with scattered fibres resembling thin collagen fibrils, each having a diameter of 21.5 ± 3.7 nm, which is significantly less (*p* < 0.00001) than the diameter of the dermal corneal collagen fibrils (28.4 ± 4.0 nm). When viewed using scanning electron microscopy, these fibrils appear to be accumulated mainly on the posterior surface of the basement membrane of the central region of the dermal cornea, as shown in Figures 6A,B, although there are fewer fibrils seen adhering to the basement membrane in the peripheral region of the dermal cornea (Figures 6C,D).

### Scleral Cornea

**FIGURE 7:**
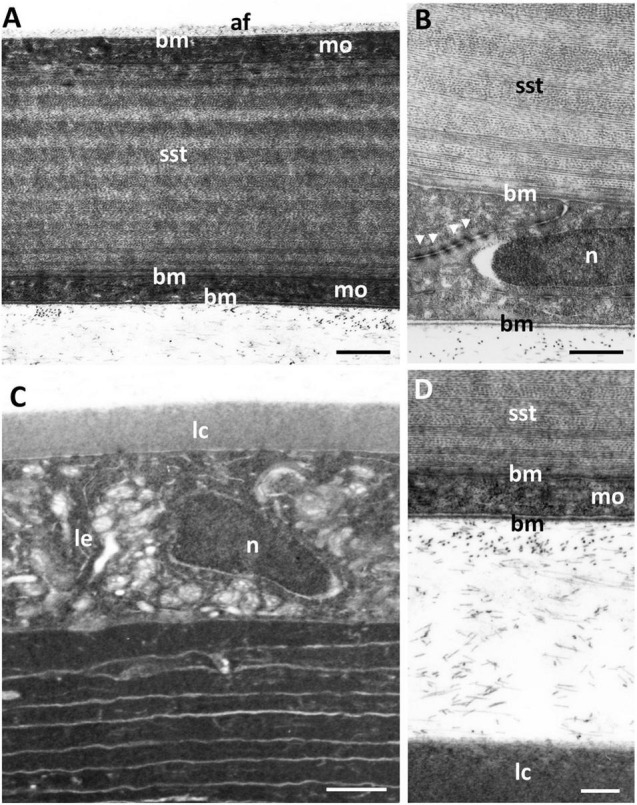
Transverse section of the scleral stroma and the anterior region of the lens. **(A)** The scleral cornea is bounded by monolayer of cells (mo), with the anterior monolayer covered by a basement membrane (bm) and the posterior monolayer (or endothelium) surrounded by basement membranes on both sides. The scleral stroma (sst) comprises collagen lamellae. The scleral cornea is separated from the lens by scattered fibres. The anterior lens is bordered by a capsule (lc) and an epithelial cell layer (le), which surround the concentric layers of lens cells. **(B)** Posterior of the scleral cornea showing the anterior and posterior basement membranes bordering two opposing endothelial cells linked by tight junctions (arrowheads). **(C)** Scattered fibres between the scleral cornea and lens. n, nucleus. **(D)** The anterior chamber between the posterior scleral cornea and the lens capsule, showing the scattered collagen fibrils. Scale bars: 2 μm **(A)**; 0.3 μm **(B)**; 1.5 μm **(C)**; 0.7 μm **(D)**.

The scleral cornea is continuous with the sclera of the globe and has a thickness of 6.58 μm at the centre and 17.83 μm in the periphery (Table 1 and Figures 1G, 7A). The scleral stroma is 4.75 ± 0.64 μm thick in the centre and 15.6 ± 4.5 μm in the periphery and contains 19 or 20 lamellae of collagen fibrils with each lamella oriented at right angles to the adjacent lamellae (Figure 7A). The collagen fibrils have a diameter of 24.2 ± 1.2 nm, which is significantly different from the collagen in the dermal cornea (*p* < 0.00001) and mucoid layer (*p* = 0.0022). No cells, i.e., keratocytes or inflammatory cells, are present within the stroma of the scleral cornea. No branching and anastomosing of collagen lamellae or any vertical sutures are present in the scleral cornea (Collin and Collin, 2021b).

The scleral stroma is bounded by two monolayers of cells, one anteriorly and one posteriorly, where the posterior layer may be considered to be a corneal endothelium. On the mucoid side of the anterior monocellular layer, which has a thickness of 978 ± 29 nm, there is a continuous basement membrane with a thickness of 25 ± 3 nm (Figure 6E) that is covered in dense fibrils (Figure 6F). There is no basement membrane on the stromal side of the anterior monolayer. The posterior monocellular layer (707 ± 45 nm thick) of the scleral stroma has a well-formed continuous basement membrane, with a thickness of 37 ± 12 nm, on the posterior (anterior chamber) side and a more diffuse basement membrane with a thickness of 86 ± 15 nm on the stromal side (Figures 7A,B). The basement membrane (on the stromal side) does not represent a true Desçemet’s membrane, as it does not have any obvious structure and there is no periodic banding. No microvilli or cilia are present extending into the anterior chamber but loose amorphous material and fibres are located in between the scleral cornea and the lens (Figures 7C,D). A summary of the dimensions of the corneal components is presented in Table 1.

### Annular Ligament

**FIGURE 8:**
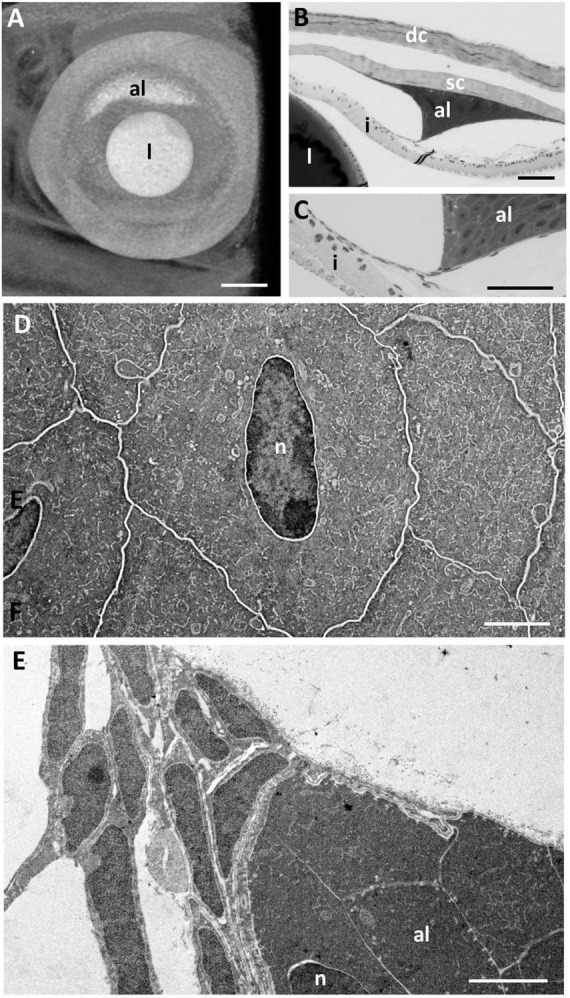
The annular ligament. **(A)** Micro-computed tomography image of the eye of *G. australis* clearly showing the location of the dorsal annular ligament (al). Rostral is toward the left. **(B,C)** Light micrographs of the anterior chamber of the eye showing the triangular annular ligament confluent with the posterior edge of the scleral cornea **(B)** and tapering and joining the anterior iris **(C)**. **(D)** Electron micrograph showing the ultrastructure of the cells comprising the annular ligament with darkly-stained cytoplasm but cell membranes that are not osmiophilic. **(E)** Tapering of the annular ligament as it projects centrally and joins the iris [as in panel **(C)**]. dc, dermal cornea; i, iris; l, lens; n, nucleus; sc, scleral cornea. Scale bars: 0.5 mm **(A)**; 0.1 mm **(B)**; 0.1 mm **(C)**; 2.5 μm **(D)**; 2.5 μm **(E)**.

In the periphery of the scleral cornea, between the basement membrane of the scleral stroma and the corneal endothelium (posterior cellular layer) is an “annular ligament” (Figures 1G, 8). However, the “annular ligament” is not truly annular or ligamentous, since it is only well-developed dorsally, tapering laterally (rostrally) to be much thinner (and even non-existent) caudally and ventrally (Figure 8A). It is triangular in cross-section being thickest (up to 135 μm) in the peripheral cornea and tapering to the angle of the anterior chamber for a distance of up to 500 μm and also tapering toward the central cornea, for up to 300 μm, terminating well before reaching the central cornea. At its peak thickness, the annular ligament spans the anterior chamber angle and is reflected onto the anterior surface of the iris (Figures 8B,C,E). The annular ligament consists of large cells, 4.2–29.1 μm in their longest dimension (mean 13.84 ± 5.01 μm) with large oval nuclei (Figure 8D). The majority of the cytoplasm is filled with a darkly-staining (osmiophilic) amorphous material, with few organelles. When viewed in both fixed and living animals, the annular ligament is thickest and most well-developed i.e., dense, when viewed using micro-computed tomography (Figure 8A) in the dorsal region of the eye, “filling” or overlying the gap produced by a dorsal notch in the iris, where a region of irideal tissue is deflected dorsally (Figures 1B,C,E,F).

At the corneal apex of the annular ligament, the posterior monocellular layer (endothelium) of the scleral cornea becomes duplicated. The endothelial cells maintain some of their characteristics, in that the nuclei are irregular compared with the oval-shaped nuclei of the “ligament” cells, the cytoplasm is more lightly-stained, there are more organelles and the cells are attached to each with occasional desmosomes. Desmosomes are not present between the cells of the annular ligament, which are frequently separated by large spaces. An additional feature is that the cell membranes of the endothelial cells stain black (osmiophilic) using the methods indicted, while the cell membranes of the annular ligament are not osmiophilic, where the cell borders appear as white lines (Figures 8D,E). About 200 μm from its corneal origin, the endothelial cells stop, although the basement membrane is still present on the anterior chamber side of the annular ligament cells.

At the apex of the triangular annular ligament nearest the iris, there are additional cells, each of which is separated from the annular ligament cells and from each other by a basement membrane. These cells resemble endothelial cells, in that they have large elongated nuclei and very little cytoplasm (Figure 8E), in marked contrast to the cells of the annular ligament, which are predominantly composed of cytoplasm (Figure 8D). These cells continue along the surface of the iris for up to 200 μm, as double or multiple layers of cells with an irregular basement membrane on both sides. Endothelial cells are also present on the surface of the iris peripheral to the annular ligament extending toward the anterior chamber angle (Figures 8B,C).

In some specimens, there is a large mass of cellular tissue (up to 175 μm long and 30 μm wide), which extends within the anterior chamber from the apex of the annular ligament toward the pupil. It appears to be present where the “annular ligament” is well developed, although its dimensions vary. In the vicinity of these masses, the extensions of the annular ligament onto the iris surface appear to be missing, indicating that these masses may represent artefactual separation of the annular ligament tissue from the surface of the iris.

Within the anterior chamber angle, there are also some strands of cellular tissue forming a loose network attached to the annular ligament and to the trabecular meshwork. These may be related to the pectinate ligament described by other authors, although since they are attached to the annular ligament, they may be additional extensions of that structure. However, some of this network may be due to an artefactual separation of the annular ligamentous tissue from the iris surface (Figure 8B).

### Iris

**FIGURE 9:**
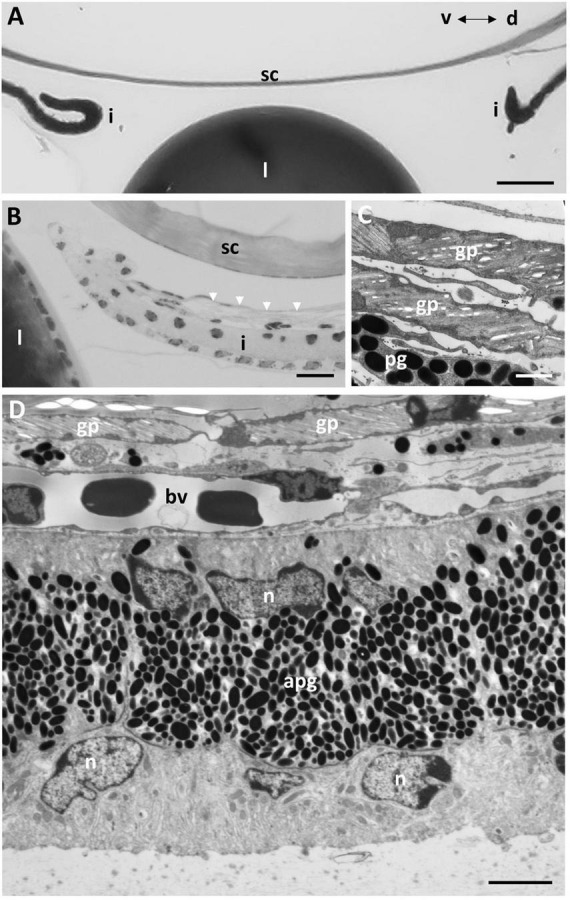
Iris structure. **(A)** Light micrograph of the anterior chamber showing the lens (l) and scleral cornea (sc) and the two limits of the dorsal and ventral profiles of the iris (i). Note the split end of the dorsal iris and the outward curve in the ventral iris. **(B)** Light micrograph showing the dorsal limit of the iris and the anterior location of the guanine plates shown in C (arrowheads). **(C)** Electron micrograph of the guanine plates (gp) covering the anterior iris with scattered pigment granules (pg). **(D)** Electron micrograph of the iris in transverse section showing the anterior guanine plates, a layer of blood vessels (bv) within the fibrovascular stroma and the dense anterior aggregation of pigment granules (apg) within the peripheral region of the iris. The posterior epithelial cells are devoid of pigment granules in the peripheral iris. n, nucleus. Scale bars: 0.1 μm **(A)**; 8 μm **(B)**; 8 μm **(C)**; 30 μm **(D)**.

In life, the iris appears silvery, with a non-mobile, slightly asymmetric pupil with the ventral radius of curvature of the iris being only two-thirds of the dorsal radius (Figures 1A,B,D,E). The superior margin of the pupil has two indentations or notches, giving rise to a small and bifurcated flap (or operculum) with extensions anteriorly (70 μm) and caudally (55 μm), that is deflected dorsally to lie approximately perpendicular to the plane of the iris (Figures 1C,F, 9A). Structurally, the iris has an anterior single epithelial cell layer, a fibrovascular stroma with large blood vessels and two posterior layers of pigment epithelium (Figures 9B–D). The fibrovascular stroma is around 18 μm thick in the periphery, tapering to a thickness of 2–3 μm near the pupil. The anterior pigment epithelial layer (posterior to the stroma) consists of large densely-pigmented cuboidal cells, 17.9–20.5 μm thick in the periphery and 10.3–12.0 μm centrally. The posterior pigment epithelial layer has a thickness of approximately 7.7–8.5 μm peripherally (and is non-pigmented) and 5.5–6.1 μm more centrally, where it contains scattered pigment granules (Figure 9D). Aggregations of stacks of what may have contained guanine crystals are located in the anterior of the iris, which would give rise to the silvery appearance of the iris in life (Figures 9C,D).

## Discussion

### The Evolution of Lamprey Cornea

The eyes of humans, mammals and birds have only one cornea, while some other vertebrates possess two corneas. A primary spectacle (or dermal cornea) and a scleral cornea are found in lampreys (Walls, 1942), including, *G. australis* (this study), the Sea lamprey, *Petroymyzon marinus* (Van Horn et al., 1969a,b; Pederson et al., 1971), the European river lamprey, *Lampetra fluviatilis* (Dickson and Graves, 1981; Dickson et al., 1982) and tadpoles and aquatic adult amphibians (Walls, 1942). The formation of a primary spectacle occurs when the superficial layers of the cornea derived from the surface ectoderm (primary spectacle) do not fuse with the deeper layers of mesodermal origin (scleral cornea) (Pederson et al., 1971). A similar arrangement may also occur in bottom-dwelling fishes, where dermal and scleral corneas have been reported, i.e., in the Salamanderfish *Lepidogalalaxias salamandroides* (Collin and Collin, 1996) and the Pipefish, *Corythoichthyes paxtoni* (Collin and Collin, 1995) and both mudskippers (*Periophthalmus* spp.) and lungfishes (*Protopterus* spp.) (Walls, 1942). However, the corneal arrangement in these species may be considered to be secondary spectacles, which occur when a transparent area of the lower lids develops or when there is fusion of the upper and lower lids, forming a true cavity lined with epithelial cells, as found in some fishes and reptiles (Franz, 1934; Walls, 1942). However, further work is needed to differentiate the embryological origins of primary and secondary spectacles in fishes.

### The Structure and Function of Corneal Surface Holes

The surface holes in the epithelium of the dermal cornea of *Geotria australis* appear to be a characteristic feature in high abundance with an inverse relationship between the size of the holes and the size of the epithelial cells. The presence of these holes has been previously reported for the Pouched lamprey *Geotria australis* (Collin and Collin, 2000a,^2006^), the Shorthead lamprey, *Mordacia mordax* (Collin and Collin, 2006), the ammocoete stage (Dickson et al., 1982), but not the adult stage, of the Sea lamprey (*Petromyzon marinus*) (Van Horn et al., 1969a; Pederson et al., 1971; Dickson et al., 1982), the Black shark, *Dalatias licha* (Collin and Collin, 2000b,^2006^), and the Australian lungfish, *Neoceratodus forsteri* (Collin and Collin, 2006). Surface holes also occur in the pre-metamorphic Axolotl, *Ambystoma mexicanum* (Collin and Collin, 2000b,^2006^, 2021a) but have almost completely disappeared in the post-metamorphic stage (Collin and Collin, 2021a). The inverse correlation between the surface cell diameter and the width of the holes found in the Pouched lamprey has also been reported for the pre-metamorphic axolotl, *A. mexicanum* (Collin and Collin, 2021a). A statistical comparison of the diameter of the microholes in the two species reveals that the small, medium and large epithelial cells in *G. australis* possess microholes with a diameter of 457 ± 89 nm, 317 ± 55 and 196 ± 38 nm, respectively. A similar relationship exists for the axolotl, where the small, medium and large epithelial cells in *A. mexicanum*, possess microholes with a diameter of 597 ± 212 nm, 400 ± 184 nm and 187 ± 81 nm, respectively.

The presence of high numbers of cytoplasmic vacuoles aggregated on the superficial side of the wing cells of the dermal epithelium has been reported in both the ammocoete larva (Dickson et al., 1982) and the adult (Van Horn et al., 1969a) of the Sea lamprey, *P. marinus*. However, the slender mucus-filled channels containing membrane-bound vesicles are present in the superficial epithelial cells of the ammocoete Sea lamprey (Dickson et al., 1982) but are not found in the adult animal (Van Horn et al., 1969a). From this ultrastructural study on adult *G. australis*, it is now apparent that the wing cells migrate to the surface of the dermal cornea, where they appear as small cells and release their mucus-filled vesicles through large holes. As the cells spread out onto the surface and the majority of the vesicles have been released, the holes become smaller. When the cytoplasm is eventually devoid of mucus vesicles, the cells become large and flattened and the holes disappear as a prelude to the cell being sloughed off. In humans, the cell turnover is about 7 days (Edelhauser et al., 1994) but the turn-over time is unknown in *G. australis*. The mucus protects the surface of the corneal epithelium, especially as a barrier to pathogens, helps maintain hydration of the ocular surface and is an important component of the corneal tear film (Hodges and Dartt, 2013).

### Differences in Corneal Thickness

The thickness of the central cornea of downstream adult *Geotria australis* was previously measured by Collin et al. (1999) and was reported to be 120 ± 20 μm, which is much greater than our combined finding of a thickness of 56.43 μm in this study. However, their measurements were made on unfixed, fast-frozen eyes (with no histological shrinkage) and included the dermal and scleral corneas including the mucoid layer, the thickness of which we were unable to accurately assess. The difference between these two measurements (63.57 μm) may be an indication of the central thickness of the mucoid layer. The central thickness of the cornea (dermal plus scleral plus mucoid layer) of another southern hemisphere lamprey (*Mordacia mordax*) is 240 μm (Collin and Potter, 2000). It is difficult to compare the central thickness of the dermal cornea of *G. australis* (49.85 μm) with other species, which do not have a primary spectacle. However, the structure of the primary spectacle (dermal cornea) of the *G. australis* is similar to that of the secondary spectacle of snakes, in that there is an epithelium, stroma and endothelium (Da Silva et al., 2016), but among 14 species of snakes, the central thickness of the stroma varies from 9.0 to 132.3 μm, compared with 24.4 μm for the *G. australis*.

The greater thickness of the dermal cornea in the periphery (173.05 μm) compared with the centre (49.85 μm) in *G. australis* appears not to have been reported in other lampreys, but is common in jawed fishes including the Florida garfish, *Lepisosteus platyrhincus*, in which the dorsal (390 μm) and ventral (310 μm) limbal corneal thicknesses are greater than the central thickness (240 μm) (Collin and Collin, 1993). In mammals and most other vertebrates, the cornea is thin in the centre compared with the periphery, which is approximately 50% thicker (Gipson, 1994). Known exceptions are the Sandlance *Limnichthyes fasciatus*, in which the central thickness is more than four times that of the periphery, primarily due to the presence of a refractive autochthonous layer (Collin and Collin, 1988; Pettigrew and Collin, 1995) and both the Trout, *Salmo fario* (Tripathi, 1974) and the Pipefish *Corythoichthyes paxtoni* (Collin and Collin, 1995), in which the centre thickness is roughly twice that of the periphery. The epithelium of the dermal cornea in *G. australis* is 5–6 layers deep, which is similar to *P. marinus* (4–5 layers) (Van Horn et al., 1969a) and *L. salamandroides* (4–5 layers) (Collin and Collin, 1996) but more than *C. paxtoni* (2 layers) (Collin and Collin, 1995) and less than *L. platyrhincus* (10 layers)(Collin and Collin, 1993). Epithelial thickness is a function of the level of corneal transparency and protection required, both of which would be vital for vision in brightly-lit, shallow water and repeated dives into the substrate during the downstream phase of the lifecycle of *G. australis*.

### The Lack of Bowman’s Layer

Specialisation of the anterior corneal stroma into Bowman’s layer with randomly-arranged collagen fibrils occurs in humans and primates (Collin and Collin, 2001), although its appearance in other species of vertebrates appears to be somewhat random (Wilson, 2020). Bowman’s layer was not observed in the dermal cornea of the Pouched lamprey *Geotria australis* (this study), and both the Sea lamprey, *Petromyzon marinus* and the European river lamprey, *Lampetra fluviatilis* (Rochon-Duvigneaud, 1943). However, this is in contrast to the findings of Van Horn et al. (1969a), who described a Bowman’s layer in the dermal cornea of *P. marinus*. It has been reported in some elasmobranchs, i.e., Spiny dogfish (*Squalus acanthias)* (Goldman and Benedek, 1967; Alanazi et al., 2015), the Stingray, *Dasyatis Americana* (10–20 μm thick, Alanazi et al., 2015) and the Clearnose skate, *Raja eglanteria* (Conrad et al., 1994) and some species of teleosts, i.e., the Brown trout (*Salmo trutta*), the Brook trout (*Salmo gairdneri*) and the Rainbow trout (*Salvelinus fontinalis*) constituting about 12.5% of the stroma (Edelhauser and Siegesmund, 1968). However, the collagen fibrils of Bowman’s layer in all these species appear to be oriented in a horizontal pattern and are not randomly distributed as observed in mammals (Edelhauser and Siegesmund, 1968). Although the function of Bowman’s layer is unknown these randomly-oriented collagen fibrils within the anterior region of the corneal stroma fail to modulate the passage of moderate- to large-sized proteins and therefore signify a reduction in barrier function (Wilson, 2020). It is unknown why this is important in *G. australis*.

### The Dermal Stroma and the Structure and Function of Vertical Sutures

The stroma of the dermal cornea of the Pouched lamprey *Geotria australis* has a number of specialised features. In addition to the absence of a Bowman’s layer, the anterior lamellae are very thin (0.047 μm) and comprise only one collagen fibre, compared with the central (0.337 μm) and posterior (0.100 μm) lamellae. A similar variation has been shown in several species of vertebrates (Table 3) but in the majority, the central lamellae are the thickest, while in humans, the opposite is true. The anterior lamellae of *G. australis* consist of only one collagen fibril, which is much narrower than all other reported species of vertebrates except the Pipefish, *Corythoichthyes paxtoni* (3 collagen fibrils) (Collin and Collin, 1995). The diameter of the collagen fibrils has been reported in many aquatic species and all fall within the range between 17 and 40 nm and, although some of these differences may be due to a range of processing techniques, regional differences in collagen fibril diameter are common within a species, i.e., dermal versus scleral stromal lamellae (Table 4). In snakes, the thickness of the collagen lamellae appears constant (Da Silva et al., 2016). Differences in collagen fibril diameter are directly related to the mechanical properties of the tissue, whereby large collagen fibrils are predicted to have a greater tensile strength and small diameter fibrils increase surface area and improve the probability of interfibrillar cross links between collagen fibrils and the components of the matrix (Parry et al., 1978).

**TABLE 3:** The thickness of collagen lamellae in different regions of the stroma of the dermal cornea in *Geotria australis* compared to the corneas of other vertebrates.

Species	Anterior stroma	Middle stroma	Posterior stroma	Number of lamellae	References
Pouched lamprey *Geotria australis*	0.047 μm	0.337 μm	0.100 μm	100	This study
Stingray *Dasyatis americana*	2.62 μm	8.65 μm	4.80 μm	25	Alanazi et al., 2015
Stingray *Dasyatis americana*	7.6 μm	8.9 μm	5.2 μm		Alibrahim et al., 2011
Spiny dogfish *Squalus acanthias*	12.14 μm	18.3 μm	13 μm		Alibrahim et al., 2011
Spiny dogfish *Squalus acanthias*	1.456 μm	5.82 μm	5.535 μm	24–25	Goldman and Benedek, 1967
Dhub lizard *Uromastyx aegyptia*	0.36 μm	1.47 μm	0.79 μm		Akhtar et al., 2016
Florida garfish *Lepisosteus platyrhinchus*	∼3.0 μm	∼3.0 μm	0.20 μm	55–65	Collin and Collin, 1993
Humans *Homo sapiens*	1.75 μm	0.68 μm	2.63 μm	242	Bergmanson et al., 2005

*The totals are the sum of the components for the dermal and scleral corneas.*

**TABLE 4:** Collagen fibril diameter in the cornea of a range of aquatic vertebrates.

Species		Region	Fibril diameter	Source
Pouched lamprey	*Geotria australis*	Primary spectacle	28.5 ± 4 nm	This study
		Mucoid layer	23.5 ± 4.0 nm	This study
		Scleral cornea	24.2 ± 1.2 nm	This study
Sea Lamprey	*Petromyzon marinus*	Scleral cornea	39.26 nm	Van Horn et al., 1969a
Dogfish	*Koinga lebruni*	Cornea	25.2 nm	Craig and Parry, 1981
Elephant fish	*Callorhynchus milii*	Cornea	25.5 nm	Craig and Parry, 1981
Stingray	*Bathytoshia brevicaudata*	Cornea	24.5 nm	Craig and Parry, 1981
Stingray	*Dasyatis americana*	Cornea	22.13 nm	Alanazi et al., 2015
Shark	*Squalis acanthias*	Cornea	24.25 nm	Alanazi et al., 2015
Shark	*Squalis acanthias*	Cornea	26.9–32.7 nm	Goldman and Benedek, 1967
Salamanderfish	*Lepidogalaxias salamandroides*	Secondary spectacle	30–40 nm	Collin and Collin, 1996
Pacific tomcod	*Microgadus proximus*	Dermal cornea	26 ± 4 nm	Collin and Collin, 1998
		Ant. scleral cornea	22 ± 5 nm	Collin and Collin, 1998
		Post. scleral cornea	22 ± 5 nm	Collin and Collin, 1998
Rattail	*Nezumia aequalis*	Dermal cornea	20 ± 3 nm	Collin and Collin, 1998
		Ant. scleral cornea	25 ± 3 nm	Collin and Collin, 1998
		Post. scleral cornea	20 ± 3 nm	Collin and Collin, 1998
Armoured grenadier	*Coryphanoides amartus*	Dermal cornea	27 ± 6 nm	Collin and Collin, 1998
		Ant. scleral cornea	22 ± 6 nm	Collin and Collin, 1998
		Post. scleral cornea	21 ± 2 nm	Collin and Collin, 1998
Pipefish	*Corythoichthyes paxtoni*	Dermal corneal	17–20 nm	Collin and Collin, 1995
		Scleral corneal	22–24 nm	Collin and Collin, 1995
Florida garfish	*Lepisosteus platyrhincus*	Cornea	20–40 nm	Collin and Collin, 1993

Our findings of extensive branching and anastomosing of collagen lamellae in the spectacle of the Pouched lamprey, *Geotria australis* is not consistent with the claims that branching is comparatively rare in fishes (Winkler et al., 2015) and that there is a progressive increase in the branching of the collagen lamellae moving from lowest in fishes, increased in amphibians, higher in reptiles and highest in birds (Winkler et al., 2015; Koudouna et al., 2018). Branching of lamellae is present in the central region of the dermal cornea of the Sea lamprey *Petromyzon marinus* (Van Horn et al., 1969a), however, in *G. australis*, the branching is almost entirely in the peripheral cornea, which is consistent with reports of branching in the skin of the Sea lamprey *P. marinus* (Van Horn et al., 1969a). Branching is also present in the Holostei, i.e., the Sturgeon, *Acipenser sturio*, the Chondrichthyes, i.e., the Great white shark *Carcharodon carcharias* (Koudouna et al., 2018), and the Teleostei, i.e., the Trout *Salmo trutta* (Edelhauser and Siegesmund, 1968), the Pacific tomcod, *Microgadus proximus*, the Rattail, *Nezumia aequalis* and the Armoured grenadier, *Coryphanoides (Nematonurus) armatus* (Collin and Collin, 1998). The function of branching and anastomosing may be to modulate the shear strength of the cornea (Smolek and McCarey, 1990) and to stabilise the corneal shape necessary for the development of a refractive lens (Winkler et al., 2015). As branching and anastomosing are common in birds, which move between different aerial atmospheric pressures and withstand increased pressure on the cornea due to high speed diving, and aquatic vertebrates, which move between different hydrostatic pressures associated with different depths of the water column, it is appropriate that their corneas should have additional structural adaptations compared with terrestrial vertebrates. In mammals and humans, branching is restricted to a single plane in the superficial layers of the cornea (Winkler et al., 2015), which is associated with Bowman’s layer (Winkler et al., 2011).

Four structural subtypes of vertical sutures are described in the Pouched lamprey *Geotria australis*, showing a heightened level of species-specific complexity not yet observed in the vertebrate cornea. Considered to be an adaptation for maintaining corneal transparency by inhibiting stromal swelling as a result of extreme cold (Fisher and Zadunaisky, 1977), exposure to large changes in pH (Christianson, 1982) and/or when moving between freshwater and seawater (Conrad et al., 1981; Wheaton and Edelhauser, 1983; Menasche et al., 1988; Collin and Collin, 2001), vertical sutures consist of collagen fibrils, which perpendicularly traverse stromal collagen lamellae. Vertical sutures are predominantly found in the dermal stroma in species possessing a primary spectacle, which may be associated with maintaining optical transparency in the protective “goggle” directly exposed to changing environmental conditions. This would appear to be critical for *G. australis*, which is anadromous, moving between freshwater and seawater and back to freshwater during its protracted lifecycle, and spends much of its adult marine phase in the cold waters off South Georgia (Potter et al., 1979; Prince, 1980). Sutures have been described previously in the stroma of the spectacle of the Sea lamprey *Petromyzon marinus* (Van Horn et al., 1969a,b; Pederson et al., 1971), which appear to be of our Type 4. It is unknown how these vertical sutures develop but they may originate as extensions of the fibroblasts (keratocytes), running across lamellae (Dickson and Graves, 1981; Dickson et al., 1982), which are in the process of producing the vertically arranged collagen fibrils.

Thick vertical sutures (Type 4 in this study) are also found in chondrichthyans i.e., the Spiny dogfish *Squalus acanthias* (Goldman and Benedek, 1967; Conrad et al., 1981; Alanazi et al., 2015), the Stingray *Dasyatis americana* (Alanazi et al., 2015) and the Clearnose skate, *Raja eglanteria* (Conrad et al., 1994) and elephant shark, *Hydrolagus colliei* (Collin and Collin, 2001) and teleosts i.e., Salamander fish *Lepidogalaxias salamandroides* (Collin and Collin, 1996) and the deep-sea teleost *Cataetyx laticeps* (Collin and Collin, 2001), in which they may traverse as many as ten adjacent collagen lamellae or span the full depth of the cornea, originating in the basal lamina of the epithelium and terminating in Desçemet’s membrane. Vertical sutures do not appear to be present in terrestrial species, including snakes, birds and mammals (Da Silva et al., 2016).

### The Mucoid Layer

The mucoid layer between the dermal and scleral corneas, consisting of amorphous material with scattered fibres resembling thin collagen fibrils in *Geotria australis*, appears to be a common feature in all vertebrates with split corneas. Thought to allow the eye to rotate beneath the protective goggle provided by the dermal cornea (which is continuous with the surrounding skin), the collagen fibrils appear to be very dense centrally, providing a heightened level of adherence, thereby enabling the action of the caudal cornealis muscle in retracting the lens to be more effective and accounting for the variation in the thickness of this layer centro-peripherally. The findings of endothelial basement membranes on both surfaces facing the mucoid layer and, in particular, on the anterior chamber side of the scleral corneal endothelium are very unusual features of *G. australis*, which are shared by the Sea lamprey *Petromyzon marinus* (Van Horn et al., 1969a; Dickson et al., 1982).

### Desçemet’s Membrane and an Endothelial Basement Membrane

The Pouched lamprey *Geotria australis* does not possess a Desçemet’s membrane but has a diffuse basement membrane with a thickness of 86 ± 15 nm situated between the stroma and the posterior endothelium of the scleral cornea. This is in agreement with the finding of Rochon-Duvigneaud (1943), who found a non-homogenous layer between the stroma and posterior endothelium of the scleral cornea of both the Sea lamprey *Petromyzon marinus* and the European river lamprey *Lampetra fluviatilis* but not a Desçemet’s membrane. A Desçemet’s membrane was also found to be lacking in *P. marinus* by Van Horn et al. (1969a) and Pederson et al. (1971), where only a thin, discontinuous layer of electron dense material was found to separate the endothelial cells from the stroma.

A Desçemet’s membrane has been claimed to be present in the elasmobranch cornea i.e., in the Stingray *Dasyatis americana* (Goldman and Benedek, 1967; Alanazi et al., 2015), the Spiny dogfish *Squalus acanthias* (Alanazi et al., 2015), where it is described as very fine, loosely-woven microfibrils (Alanazi et al., 2015) or as a very thin (0.3–4.4 μm) homogeneous layer of fine fibrils (Goldman and Benedek, 1967), and the teleost cornea i.e., in the Pipefish *Corythoichthyes paxtoni* (2 μm thick, Collin and Collin, 1995), the Zebrafish *Danio rerio* (0.15 μm, Zhao et al., 2006), the Salamanderfish, *Lepidogalaxias salamandroides* (∼75 to 100 nm in centre and ∼200 nm in the periphery, Collin and Collin, 1996) and in three species of deep-sea fishes (0.1–0.23 nm, Collin and Collin, 1998). However, in a number of species of trout, Edelhauser and Siegesmund (1968) found only a poorly-defined endothelial basement membrane.

It is apparent that differentiating Desçemet’s membrane from a normal endothelial basement membrane can be difficult and should not be based only upon the thickness of the membrane. In humans, Descemet’s membrane continues to grow throughout life and has a thickness of around 3 μm at birth (Murphy et al., 1984) and may reach a thickness of up to 12 μm in adults (Gipson, 1994) or even 18 to 19 μm in the elderly (Murphy et al., 1984). It has a structure with an anterior (embryonic) banded portion with a periodicity of between 110 and 120 nm (Murphy et al., 1984; Gipson, 1994) and a less dense, non-banded portion. A similar structure has been reported in the aquatic Little penguin (*Eudyptula minor*) (Collin and Collin, 2021b) and in numerous mammals, including, the rat (3.5 μm thick with a periodicity of 120 nm), the mouse (ddY strain) (2.5 μm thick with a periodicity of 120 nm), the mouse (C3H strain) (5 μm thick with a periodicity of 120 nm), the guinea pig (13 μm thick with a periodicity of 120 nm), the cat (periodicity of 100 nm), the rabbit (5.6 μm thick) and various species of cattle (20 μm thick, periodicity 120 nm) (Hayaski et al., 2020).

Desçemet’s membrane or the posterior elastic lamina has been described as the basement membrane of the corneal endothelium (Gipson, 1994) or as a dense, thick, relatively transparent and cell-free matrix that separates the corneal stroma from the endothelium (de Oliviera and Wilson, 2020). Murphy et al. (1984) describe the three major processes, necessary to form a Desçemet’s membrane; namely growth in the prenatal period, differentiation into a striated basement membrane and an increase in thickness in the postnatal period, giving rise to an extraordinarily thick and multilayered structure. Jakus (1956) states that Desçemet’s membrane has a hexagonal structure and is banded (107 μm) in transverse section.

If we adopt the descriptions of Gipson (1994) and de Oliviera and Wilson (2020), almost all vertebrates possess a Desçemet’s membrane. However, if we adopt the definitions of Jakus (1956) and Murphy et al. (1984), all of the vertebrates with thin, incomplete, irregular collections of loosely-woven fine fibrils would be better described as having a basement membrane rather than a Desçemet’s membrane and this includes the lampreys. Further differentiation may be possible as a result of tissue analysis of the membranes, e.g., using monoclonal antibodies to label components such as collagen (Cheng et al., 2021) and laminin (Leung et al., 2000). Collagen Type IV and laminin are major components of basement membranes (Kefalides et al., 1979; Kühn et al., 1981; Sanes et al., 1990), while Desçemet’s membrane, a morphologically unique basement membrane, is rich in collagen Type VIII (Kapoor et al., 1986; Illidge et al., 2001), in addition to some collagen Type IV (Leung et al., 2000), although, in rabbits, Desçemet’s membrane does not contain laminin (Leung et al., 2000).

### The Structure, Evolution and Function of the “Annular Ligament”

The “annular ligament” of the Pouched lamprey *Geotria australis* is situated in the periphery of the scleral cornea, between the basement membrane of the scleral stroma and the corneal endothelium. Composed of large cells with amorphous cytoplasm, this triangular tissue appears to be restricted to the dorsal region of the eye in *G. australis* with its apex attached to the anterior iris. An “annular ligament” has also been described briefly in the Sea lamprey, *Petromyzon marinus* (Rochon-Duvigneaud, 1943; Dickson and Graves, 1981) and the European river lamprey, *Lampetra fluviatilis* (Walls, 1942; Rochon-Duvigneaud, 1943) but in several studies of the ultrastructure of the cornea in Sea lamprey, *Petromyzon marinus*, there is no mention of an “annular ligament” (Van Horn et al., 1969a; Pederson et al., 1971; Dickson et al., 1982) suggesting that it is also not annular in these species of lampreys. There have been no previous detailed descriptions of the “annular ligament” in any southern hemisphere lampreys.

The structure of the “annular ligament” in *G. australis* is similar to that described for what may be a homologous structure in the Goldfish *Carassius auratus*, i.e., tissue comprised of large polyhedral cells, with few mitochondria and indistinct cristae (Tripathi, 1974) and in the Zebrafish, *Danio rerio* i.e., with a cytoplasm, which contains an abundant accumulation of glycoproteins, including keratocan and lumican (Chen et al., 2008). Walls (1942) described the “annular ligament” in *L. fluviatilis* as a conspicuous thickening composed of epithelioid cells, which may represent a “piling up” of Desçemet’s mesothelium. Based on its structure and chemical composition, the term “annular ligament” appears inappropriate, as previously pointed out by Rochon-Duvigneaud (1943), who termed this the “vesioculo-hyaline tissue of the angle” and Tripathi (1974), as collagen is not present within the structure or its cellular components (Collin and Collin, 1996). Chen et al. (2008) described the “annular ligament” of *D. rerio* as a “prominent ligament-like fibrous meshwork” but then reported that the cells contained abundant accumulations of the glycoproteins, keratocan and lumican, with no mention of collagen. The “annular ligament” also lacks the characteristic alignment of parallel bundles of collagen fibrils and fibroblasts observed in the ligaments of mammals (Redler et al., 2019; Kaku et al., 2020).

The origin of the “annular ligament” has been debated but our findings indicate that it is derived from the scleral corneal endothelium, which persists until the annular ligament markedly increases in thickness. The continuation of the basement membrane without an obvious endothelium implies that the cells still maintain this membrane-producing ability in *G. australis*. From an evolutionary perspective, the “annular ligament” may have been present in the last common ancestor of lampreys given it has been retained in at least some species of Chondrichthyes (Elasmobranchii), i.e., the Dogfish *Squalus acanthias* (Tripathi, 1974), although it is not described in the eyes of the Stingray *Dasyatis americana* (Alanazi et al., 2015), the Clearnose skate *Raja eglanteria* (Conrad et al., 1994) or non-actinopterygian early ray-finned fishes including the Chondrostei. It is poorly developed in *Polypterus* spp. (Duke-Elder, 1958) and the Holostei, but particularly well-developed in the dorsal region of the cornea in the Florida garfish *Lepidosteus platyrhincus* (Duke-Elder, 1958; Tripathi, 1974; Collin and Collin, 1993) and the Teleostei, i.e., where it is common and well-developed in cyprinids (Rochon-Duvigneaud, 1943), extending across most of the surface of the iris (Oppel and Franz, 1913), a species of trout *Salmo trutta* (Edelhauser and Siegesmund, 1968), the Goldfish *Carassius auratus* (Rochon-Duvigneaud, 1943; Tripathi, 1974), the Gudgeon, *Gobio fluviatilis* (Rochon-Duvigneaud, 1943), the Salamanderfish *Lepidogalaxias salamandroides* (Collin and Collin, 1996) and the Zebrafish *Danio rerio* (Yoshikawa et al., 2007; Chen et al., 2008). The “annular ligament” appears to be absent in the Dipnoi (lungfishes) (Duke-Elder, 1958; Tripathi, 1974). The function of the annular ligament is unknown (Walls, 1942; Duke-Elder, 1958) but is suggested to be secretive (Baecker, 1931) or refractive (Collin and Collin, 1996), although due to its location in *G. australis*, it may help to support the suspension of the opercular flap in the dorsal iris (see below).

### Pectinate Ligament

Similar to the debate over the name of the “annular ligament” the term “pectinate ligament” is also thought to be misleading (Wolff, 1948). According to official gross-anatomical nomenclature, “pectinatum ligamentum” is a synonym or a substitute for the trabecular meshwork (“reticulum trabeculare”) (Simoens et al., 1996), although most veterinary ophthalmologists attribute the term pectinate ligament to the anterior-most strands of the meshwork at the iridocorneal angle, whereas the more peripheral parts of the meshwork are called the trabecular meshwork (Simoens et al., 1996).

The strands of the “pectinate ligament” are defined as being comprised of a central collagen core, surrounded by mesothelium (Walls, 1942; Allen et al., 1955; Duke-Elder, 1958). However, the loose network in the eye of the Pouched lamprey *Geotria australis* appears to be cellular, lacking a collagen core and is attached to the “annular ligament,” and thereby is thought to be an extension of that structure. Walls (1942) and Duke-Elder (1958) both claim that in the lamprey eye, delicate strands, perhaps coated with mesothelium, cross from the end of the annular ligament to the periphery of the iris, like a “pectinate ligament.” These strands are prominent in *Lampetra fluviatilis*, but practically non-existent (except superiorly) in the brook lamprey *Ichthyomyzon fossor* (Walls, 1942). The “pectinate ligament” is developed to varying degrees in different species but is absent in the selachians (Elasmobranchii) (Oppel and Franz, 1913; Duke-Elder, 1958) and rudimentary or vestigal in lower placentals and humans (Allen et al., 1955; Duke-Elder, 1958). The function of the “pectinate ligament” is unknown.

### The Iris

The pupil of *Geotria australis* is bounded by a highly-reflective, silvery iris (produced by stacks of presumably guanine crystals (Dickson and Graves, 1981), which appears to be almost circular in shape in both downstream and upstream migrants. However, along the superior margin of the iris are two indentations or notches, giving rise to a small and bifurcated flap (or operculum) that is deflected dorsally. Although other regions of the superior margin of the iris are raised slightly above the pupillary plane, the flap appears to be suspended to sit perpendicular to the pupil and parallel to the surface of the head. This is an unusual feature, which has not been observed in any other species of lamprey, although it bears some resemblance to the irideal operculum of the iris in batoids (McComb and Kajiura, 2008) and catfishes (Douglas et al., 2002; Collin, 2003). Its function is unknown but may serve to reduce bright light entering the eye from above, thereby reducing intraocular flare, given that *G. australis* spends appreciable amounts of time in the surface waters of the open ocean (in its marine phase).

## Data Availability Statement

The raw data supporting the conclusions of this article will be made available by the authors, without undue reservation.

## Ethics Statement

The animal study was reviewed and approved by The University of Western Australia Animal Ethics Committee.

## Author Contributions

All authors contributed to the design of the study, the acquisition of data, data analysis, and interpretation and writing the manuscript.

## Conflict of Interest

The authors declare that the research was conducted in the absence of any commercial or financial relationships that could be construed as a potential conflict of interest.

## Publisher’s Note

All claims expressed in this article are solely those of the authors and do not necessarily represent those of their affiliated organizations, or those of the publisher, the editors and the reviewers. Any product that may be evaluated in this article, or claim that may be made by its manufacturer, is not guaranteed or endorsed by the publisher.
